# Should the Legal Age for Tobacco Be Raised? Results From a National Sample of Adolescents

**DOI:** 10.5888/pcd14.170255

**Published:** 2017-11-16

**Authors:** Sarah D. Kowitt, Allison M. Schmidt, Allison E. Myers, Adam O. Goldstein

**Affiliations:** 1 Department of Health Behavior, Gillings School of Global Public Health, University of North Carolina at Chapel Hill, Chapel Hill, North Carolina; 2 Lineberger Comprehensive Cancer Center, University of North Carolina at Chapel Hill, Chapel Hill, North Carolina; 3 Counter Tools, Carrboro, North Carolina; 4 Department of Family Medicine, University of North Carolina at Chapel Hill, Chapel Hill, North Carolina

## Abstract

Raising the minimum age of legal access to tobacco products may reduce smoking initiation and save lives. In a national telephone survey (2014–2015), US adolescents aged 13 to 17 years (N = 1,125; response rate, 66%) were asked about raising the age of legal access to tobacco products and randomized to hear one of 3 ages (19, 20, or 21 y). Most adolescents, across all US regions, favored raising the minimum age of legal access to 19 (75.7%), 20 (80.6%), or 21 (76.4%). These supportive attitudes may be useful to tobacco prevention and control practitioners who seek to reduce tobacco use among adolescents.

## Objective

Raising the minimum age of legal access to tobacco products has potential to reduce smoking initiation and save lives (1). Increasingly, cities and states (eg, Boston, New York City, Chicago, Hawaii, California) across the country have implemented policies that raise the minimum age of legal access to tobacco products to 21, the age expected to have optimal impact (1).

Most US adults, even current smokers, have supportive attitudes toward raising the minimum age of legal access to tobacco (2). However, views about such policies among young people (the most affected age group of such a policy [1]) have yet to be measured. Measuring attitudes toward policies is important because attitudes are influential in successfully implementing new policies, enforcing regulations, and understanding unintended consequences (3,4). We therefore examined attitudes toward raising the minimum age of legal access to tobacco products among a national sample of adolescents.

## Methods

The UNC Center for Regulatory Research on Tobacco Communication conducted a national telephone survey of adolescents (aged 13–17 y) living in the United States from November 2014 through June 2015 (5). Three independent and nonoverlapping frames were used for sampling, ensuring coverage to approximately 98% of US households. The weighted sample is nationally representative of adolescents who 1) are aged 13 to 17 years, 2) live in the United States, 3) have cellular or landline telephone access, and 4) if asked, could obtain consent from a guardian for a telephone survey on tobacco use. Both informed consent from the parent or guardian and assent from the adolescent were required for all adolescent participants. The sample resulted in 1,125 interviews and a weighted response rate of 66%, which is comparable to the 2014 National Youth Tobacco Survey response rate of 73% (6). All procedures were approved by the institutional review board at the University of North Carolina at Chapel Hill.

Participants were asked if they supported the US Food and Drug Administration (FDA) raising “the age of purchase for tobacco products in all states to [age]” and were randomized to hear one of 3 ages (19, 20, or 21 y). Response options included yes (a favorable view of the policy) or no (an unfavorable view). Covariates in the analyses were sex, age, race/ethnicity, parents’ education (asked of parents), sexual orientation, smoking status, and susceptibility to smoking cigarettes (7). To measure susceptibility (7), 2 validated items were used from the original 4-item measure of adolescent smoking susceptibility developed by Pierce et al, an approach used in previous research (7,8).

Using SAS version 9.3 (SAS Institute, Inc) to account for the complex survey design and sampling weights, we entered all covariates simultaneously in a multivariable weighted logistic regression model. Respondents who reported that they did not know or had no opinion (n = 36) and/or were missing demographic information (n = 46) were dropped from the sample, which yielded a sample size of 1,043. We tabulated weighted percentages, adjusted odds ratios (ORs), and 95% confidence intervals (CIs). We set significance at an α of .05 and used 2-tailed tests.

## Results

Most adolescents (77.8%; 95% CI, 75.0%–80.5%) reported that the age of legal access to tobacco products should be raised (Table 1). This level of policy support was consistent for adolescents across all regions of the United States (Table 2).

**Table 1:** Weighted Characteristics of Survey Respondents and Percentage Who Had a Favorable View^a^ of Increasing the Minimum Age for Legal Access to Tobacco Products, Sample of US Adolescents Aged 13 to 17 Years, 2014–2015^b^

Variable	Unweighted No.	Weighted Characteristic, %^c^ (95% Confidence Interval)	Favorable View of Policy, Weighted % (95% Confidence Interval)
**Total**	1,043	100	77.8 (75.0–80.5)
**Sex**			
Male	528	52.0 (48.7–55.3)	74.2 (70.1–78.2)
Female	515	48.0 (44.7–51.3)	81.7 (78.0–85.4)
**Age, y**			
13	164	16.9 (14.3–19.4)	85.8 (80.1–91.5)
14	219	22.0 (19.2–24.8)	85.1 (79.8–90.4)
15	229	20.7 (18.2–23.5)	79.3 (73.3–85.2)
16	224	21.0 (18.3–23.6)	73.5 (67.1–79.9)
17	207	19.4 (16.8–22.0)	65.5 (58.4–72.6)
**Race**			
White	837	73.4 (70.4–76.5)	78.4 (75.4–81.4)
Black or African American	111	13.2 (10.9–15.5)	75.5 (67.0–84.0)
American Indian or Alaska Native	16	2.0 (1.0–3.0)	83.2 (65.1–100.0)
Asian	20	2.8 (1.6–4.0)	81.3 (64.1–98.5)
Pacific Islander	3	0.5 (0–1.1)	100.0 (100.0–100.0)
Other	56	8.1 (6.1–10.2)	72.0 (59.7–84.2)
**Ethnicity**			
Latino/Hispanic	75	9.3 (7.2–11.4)	73.9 (63.0–84.7)
Non-Latino/non-Hispanic	968	90.7 (88.6–92.8)	78.2 (75.3–81.0)
**Parent education**			
Less than high school	64	6.3 (4.6–8.0)	74.2 (61.8–86.6)
High school graduate	155	12.9 (10.9–15.0)	82.2 (76.0–88.4)
Some college	181	17.9 (15.3–20.6)	78.1 (71.5–84.6)
Associate degree	107	10.2 (8.2–12.1)	80.5 (72.8–88.3)
Bachelor’s degree	318	30.3 (27.3–33.3)	76.4 (71.3–81.4)
Graduate or professional degree	218	22.3 (19.5–25.2)	76.7 (70.4–82.9)
**Sexual orientation**			
Straight or heterosexual	1,003	96.1 (94.8–97.3)	78.5 (75.7–81.3)
Gay, lesbian, or bisexual	40	3.9 (2.7–5.2)	60.1 (43.6–76.6)
**Susceptibility to smoking cigarettes^d^**			
Not susceptible	854	82.6 (80.1–85.1)	83.9 (81.2–86.6)
Susceptible	151	14.3 (11.9–16.6)	51.8 (43.0–60.6)
Current cigarette smoker	38	3.2 (2.1–4.2)	35.8 (19.9–51.8)
**Age proposed by survey as legal age of access to tobacco products^e^**			
19 y	336	31.0 (27.9–34.0)	75.7 (70.7–80.6)
20 y	376	37.7 (34.4–40.9)	80.6 (76.3–84.9)
21 y	331	31.4 (28.3–34.5)	76.4 (71.3–81.6)

a Participants were asked if they supported the US Food and Drug Administration raising “the age of purchase for tobacco products in all states to [age].” Response options included yes (a favorable view of the policy) or no (an unfavorable view).

b Data source: The UNC Center for Regulatory Research on Tobacco Communication national telephone survey of adolescents.

c Percentages may not sum to 100 because of rounding.

d Adolescents who reported not smoking cigarettes in the past 30 days were asked to indicate their willingness to smoke cigarettes in the next year and to smoke cigarettes if a best friend offered one. Participants who chose anything but “definitely no” in response to the 2 questions were classified as susceptible to cigarette smoking (7).

e Participants asked if they supported the US Food and Drug Administration raising “the age of purchase for tobacco products in all states to [age]” were randomized to hear one of 3 ages (19, 20, or 21 y).

**Table 2:** Percentage of Survey Respondents Who Had a Favorable View of Increasing the Minimum Age for Legal Access to Tobacco Products, Sample of US Adolescents Aged 13 to 17 Years (Unweighted N = 1,043), by Region, 2014–2015^a^

Variable	Unweighted No.	Favorable View of Policy, Weighted % (95% Confidence Interval)^b^
**All 50 states and the District of Columbia**	1,043	77.8 (75.0–80.5)
**New England** (Connecticut, Maine, Massachusetts, New Hampshire, Rhode Island, Vermont)	44	73.3 (60.0–86.5)
**Middle Atlantic **(New Jersey, New York, Pennsylvania)	101	76.4 (67.9–84.9)
**East North Central **(Illinois, Indiana, Michigan, Ohio, Wisconsin)	168	74.5 (67.6–81.4)
**West North Central** (Iowa, Kansas, Minnesota, Missouri, Nebraska, North Dakota, South Dakota)	98	77.4 (69.1–85.8)
**South Atlantic** (Delaware, District of Columbia, Florida, Georgia, Maryland, North Carolina, South Carolina, Virginia, West Virginia)	270	80.7 (75.6–85.8)
**East South Central** (Alabama, Kentucky, Mississippi, Tennessee)	138	75.1 (67.6–82.6)
**West South Central** (Arkansas, Louisiana, Oklahoma, Texas)	98	80.2 (72.1–88.4)
**Mountain** (Arizona, Colorado, Idaho, New Mexico, Montana, Utah, Nevada, Wyoming)	66	78.9 (69.0–88.8)
**Pacific** (Alaska, California, Hawaii, Oregon, Washington)	60	79.5 (69.0–90.0)

a Data source: The UNC Center for Regulatory Research on Tobacco Communication national telephone survey of adolescents.

b We found no statistical differences in views toward the policy among US regions.

We found no significant associations between the age of legal access to which adolescents were randomly assigned and whether they supported raising the age of legal access (Table 3). Girls had higher odds than boys of having a favorable view of increasing the age of legal access (adjusted OR, 1.61; 95% CI, 1.13–2.30). Compared with adolescents classified as not susceptible to smoking cigarettes, adolescents classified as susceptible to cigarette smoking (adjusted OR, 0.22, 95% CI, 0.14–0.33) and adolescents currently smoking cigarettes (adjusted OR, 0.15, 95% CI, 0.07–0.32) had lower odds. Additionally, gay, lesbian, and bisexual adolescents (adjusted OR, 0.49, 95% CI, 0.24–0.98), compared with straight or heterosexual adolescents, and older adolescents (adjusted OR, 0.76, 95% CI, 0.66–0.87) had lower odds of a favorable view. We found no differences by race or ethnicity.

**Table 3:** Associations With Favorable Views of Increasing the Minimum Age for Legal Access to Tobacco Products, Sample of US Adolescents Aged 13 to 17 Years (Unweighted N = 1,043), 2014–2015^a^

Variable	Weighted Adjusted Odds Ratio (95% Confidence Interval)	*P* Value
**Susceptibility to smoking cigarettes^b^**		
Susceptible	0.22 (0.14–0.33)	.03
Current smoker	0.15 (0.07–0.32)	.003
Not susceptible	1.0 [Reference]	—
**Age proposed by survey as legal age of access to tobacco products^c^**		
21	1.29 (0.85–1.94)	.72
20	1.06 (0.69–1.64)	.22
19	1.0 [Reference]	—
**Respondent age**	0.76 (0.66–0.87)	<.001
**Sex**		
Female	1.61 (1.13–2.30)	.009
Male	1.0 [Reference]	—
**Race/ethnicity**		
Non-white	0.91 (0.59–1.41)	.68
Latino/Hispanic	0.82 (0.41–1.64)	.57
Non-Hispanic white	1.0 [Reference]	—
**Sexual orientation**		
Lesbian, gay, or bisexual	0.49 (0.24–0.98)	.04
Straight or heterosexual	1.0 [Reference]	—

a Data source: The UNC Center for Regulatory Research on Tobacco Communication national telephone survey of adolescents.

b Adolescents who reported not smoking cigarettes in the past 30 days were asked to indicate their willingness to smoke cigarettes in the next year and to smoke cigarettes if a best friend offered one. Participants who chose anything but “definitely no” in response to the 2 questions were classified as susceptible to cigarette smoking (7).

c Participants asked if they supported the US Food and Drug Administration raising “the age of purchase for tobacco products in all states to [age]” were randomized to hear one of 3 ages (19, 20, or 21 y).

## Discussion

In the first national study of adolescents’ attitudes toward raising the age of legal access to tobacco products, we found that more than three-quarters of US adolescents supported national efforts to raise the minimum legal age. Our findings are important for 4 reasons.

First, research shows that raising the minimum age of legal access to 21 would lead to larger reductions in tobacco use among young people than polices that raise the minimum age of legal access to 19 or 20, so it is meaningful that our data show that raising the age to 21 would be just as well received by adolescents as would potentially weaker policies, such as raising the age to 19 (1,9).

Second, young people who are susceptible to smoking (7) would likely be most affected by policies that raise the minimum age of legal access, and yet more than half of these young people supported such policies. Thus, for opponents who suggest that raising the age of legal access to tobacco products would limit individual freedom, our research finds that even adolescents who would be *most* affected by such a policy still support it.

Third, although most adolescents in almost all groups expressed favorable views toward such policies, we did observe some group differences in attitudes. These group differences can help the public health community to target communication efforts toward adolescents who are susceptible to cigarette smoking (7), adolescents who are actively smoking, and adolescents who identify as gay, lesbian, or bisexual, and may strengthen support for and compliance with such policies (10,11).

Finally, although adolescents cannot vote to enact policies to increase the minimum age of legal access to tobacco products, they can advocate for local and state policy change (eg, Hawaii [12]).

Our study has several limitations, including our inability to control for other variables that could influence the outcomes of our findings and the phrasing of our question, which referred to national efforts to raise the age of legal access to tobacco products, rather than states or localities. Despite these limitations, our study provides new data that US adolescents have supportive attitudes toward raising the minimum age of legal access to tobacco products. To prevent and reduce smoking among young people, policy makers should critically evaluate how they can best implement and enforce such a policy.
